# Preparation of Chitin Nanofibers from Mushrooms

**DOI:** 10.3390/ma4081417

**Published:** 2011-08-12

**Authors:** Shinsuke Ifuku, Ryoki Nomura, Minoru Morimoto, Hiroyuki Saimoto

**Affiliations:** 1 Department of Chemistry and Biotechnology, Graduate School of Engineering, Tottori University, 4-101 Koyama-cho Minami, Tottori 680-8550, Japan; E-Mails:s_s_a_t_school@yahoo.co.jp (R.N.); saimoto@chem.tottori-u.ac.jp (H.S.); 2 Research Center for Bioscience and Technology, Tottori University, 4-101 Koyama-cho Minami, Tottori 680-8550, Japan; E-Mail: morimoto@chem.tottori-u.ac.jp

**Keywords:** chitin, nanofibers, mushrooms

## Abstract

Chitin nanofibers were isolated from the cell walls of five different types of mushrooms by the removal of glucans, minerals, and proteins, followed by a simple grinding treatment under acidic conditions. The Chitin nanofibers thus obtained have a uniform structure and a long fiber length. The width of the nanofibers depended on the type of mushrooms and varied in the range 20 to 28 nm. The Chitin nanofibers were characterized by elemental analyses, FT-IR spectra, and X-ray diffraction profiles. The results showed that the α-chitin crystal structure was maintained and glucans remained on the nanofiber surface.

## 1. Introduction

Chitin occurs mainly in the exoskeleton of shellfish and insects and the cell wall of fungi [1]. Chitin plays the key role in their morphogenesis. A mushroom is a fleshy fungus and has a spore-bearing fruiting body. It is used as food and in medical applications. Since edible mushrooms consist mainly of chitin, glucans, and proteins present in the cell wall, they are a good source of dietary fiber. Dietary fiber is important as a functional food ingredient. It is used for food swelling, as a food thickener, film forming agent, stabilizer, and overall as an important health ingredient [2,3]. Therefore, dietary fibers of chitin are useful for improving functional foods.

**Figure 1:**
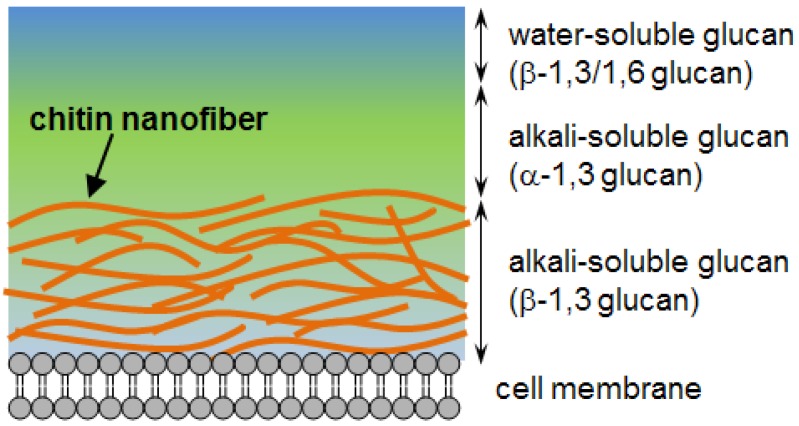
Schematic presentation of the cell-wall structure of a typical mushroom.

Recently, we have succeeded in isolating chitin nanofibers from the exoskeleton of both crab and prawn [4,5,6]. Chitin nanofibers in the exoskeletons are embedded in matrices of proteins and minerals. We isolated chitin in the form of nanofibers after removal of the matrix substances using a grinder. The obtained nanofibers have a uniform width of 10–20 nm and a long fiber length. The chitin nanofibers have a very strong potential for the development of nanofiber and chitin science and technology because of their nano-sized structure, very high surface-to-volume ratio, high water dispersibility and viscosity, excellent physical properties: high Young’s modulus and fracture strength, low thermal expansion, and above all important wound healing properties, Similarly, the cell walls of mushrooms consist of a chitin nanofiber network which is embedded in the matrices mainly of *β*-1,3 glucans as shown in Figure 1 [7].

In this study, we also applied the isolation method of chitin nanofibers from crab and prawn shells to fruiting bodies of mushrooms. However, the morphology of a mushroom cell wall is very different from that of crab and prawn shells. Highly branched *β*-1,3/1,6-glucans lie on the wall that connects the cells together since glucan mucilage is poorly organized while *α*-1,3 glucans serve as an amorphous matrix which interdigitates with the inner side of the *β*-1,3-glucan layer. The component *β*-1,3-glucans are embedded in crystalline chitin nanofibers [8]. Considering the above composition of the mushroom cell wall, we have developed a method to isolate and study the chitin in the form of nanofibers of homogeneous width from five different varieties of mushrooms. These dietary nano-sized fibers obtained from cultivable and edible mushrooms will have much scope as novel functional food ingredients.

## 2. Results and Discussion

### 2.1. Preparation of Chitin Nanofibers from Mushrooms

**Figure 2:**
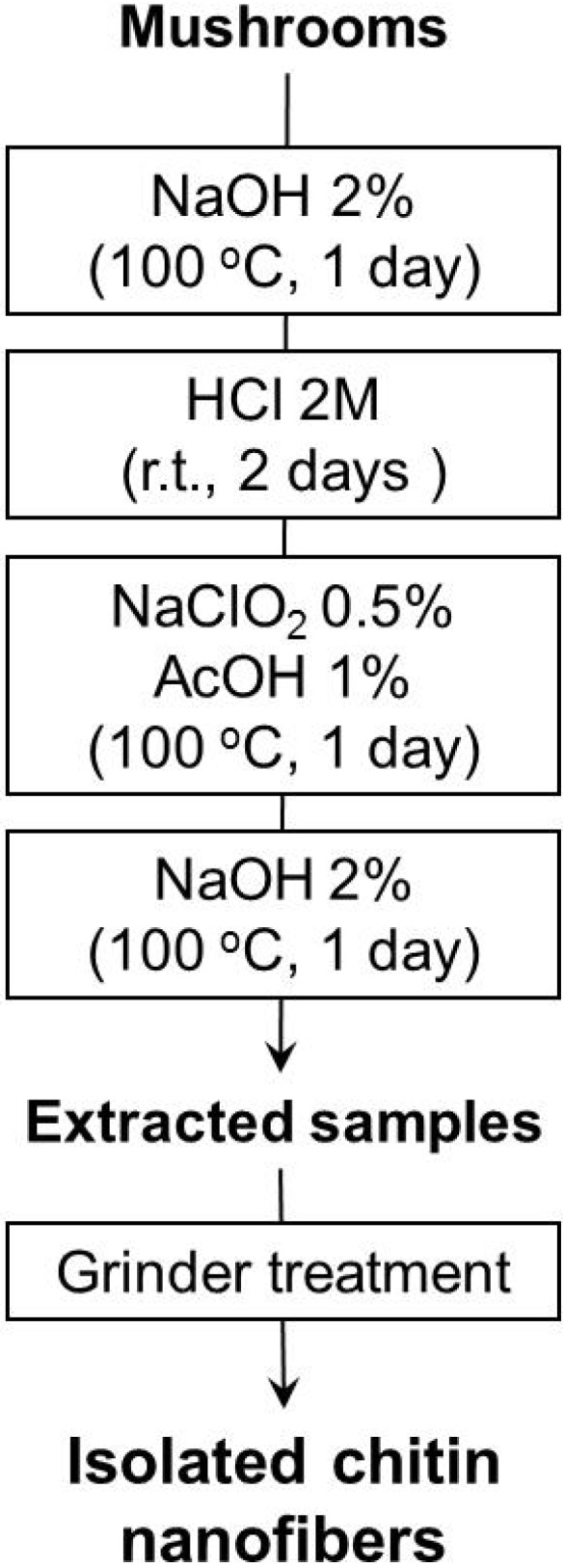
Preparation procedure of chitin nanofibers from mushrooms.

Five different species of mushrooms: *Pleurotus eryngii*, *Agaricus bisporus*, *Lentinula edodes*, *Grifola frondosa*, and *Hypsizygus marmoreus* widely used for human consumption were selected to isolate chitin nanofibers in this study. The chitin nanofibers were obtained from these mushrooms by a series of purification and chemical treatments described in Figure 2 [9] to remove associated components: proteins, pigments, glucans, and minerals. The procedure and treatments are as follows. At the first stage, sodium hydroxide was used to dissolve, hydrolyze, and remove proteins and alkali-soluble glucans. Hydrochloric acid was added to remove minerals. At this stage, it is known that partial neutral saccharides and acid-soluble protein compounds are also separated.

The extraction step with sodium chlorite and acetic acid can bleach the pigments in the sample. For the final stage, the sample was treated with sodium hydroxide again to eliminate and remove the residual glucans, including trace amounts of proteins. The drying process causes strong hydrogen bonding between chitin fibers after removal of matrix substances which makes it difficult to obtain chitin in the form of nanofibers [10]. Therefore, the sample must be kept wet after removal of the matrices to obtain chitin nanofibers. The extracted sample with 1 %w/w of chitin was passed through a grinder for nano-fibrillation along with acetic acid. Acidic conditions are necessary for nanofiber preparation. The cationization of a small portion of amino groups on the chitin fiber causes electrostatic repulsion, which facilitate nano-fibrillation. Although it is difficult to measure the degree of deacetylation (DDA) of the extracted chitin since the sample contains glucans as mentioned below, the DDA is expected to be less than 5%. See reference to the previous paper [5]. After grinder treatment, the chitin slurry had a high viscosity with a homogeneous and stable dispersibility indicating that the sample was successfully nano-fibrillated.

**Figure 3:**
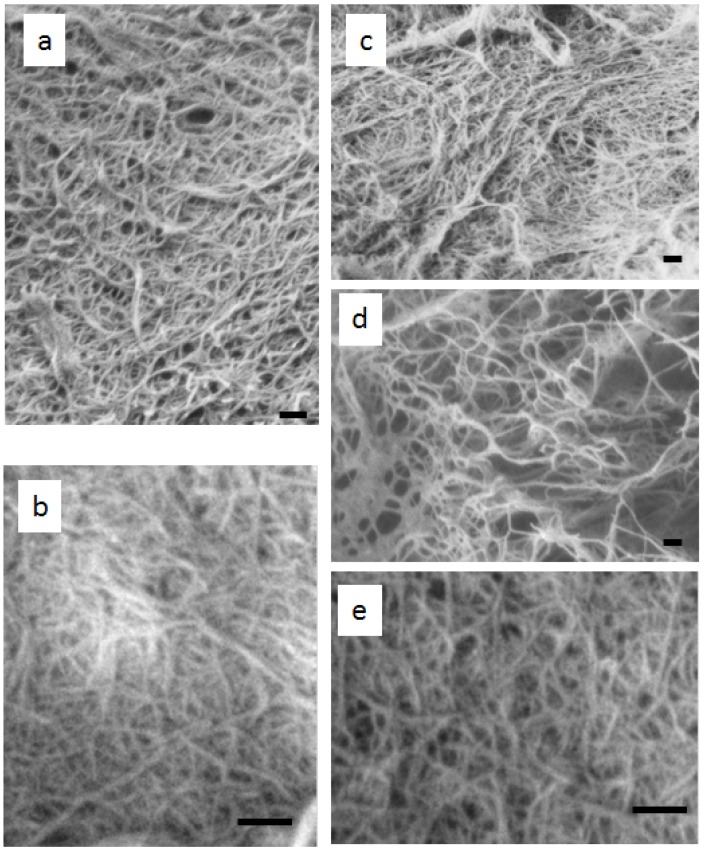
FE-SEM micrographs of chitin nanofibers prepared from (**a**) *Pleurotus eryngii* (**b**) *Agaricus bisporus* (**c**) *Lentinula edodes* (**d**) *Grifola frondosa* (**e**) *Hypsizygus marmoreus*. The scale bars are 200 nm in length.

Figure 3 shows SEM images of chitin from five mushrooms after removing matrix components and one cycle of grinding. The isolated chitins are well-fibrillated and images show uniform nanofibers. The appearance of the fibers was similar to that of chitin nanofibers prepared from crab and prawn shells. Damaged fibers were not observed during a series of chemical and mechanical treatment. The lengths of the nanofibers are very long. The average width seems slightly different depending on the type of mushroom. This will be discussed in the latter part of the text. In this study, we have demonstrated that the preparation of chitin nanofibers from several varieties of mushrooms can be done similarly to the method used for crab and prawn shell. The chitin nanofiber slurries were not transparent, which was the same situation as for the chitin nanofibers from crab and prawn shell. This is because the nanofiber surface was hydrophobic compared with cellulose nanofiber, and very long chitin nanofibers in high concentration (1%) interacted with each other.

### 2.2. Characterization of Chitin Nanofibers

The N and C contents of chitin nanofibers determined by elemental analysis are listed in Table 1. The N contents of all mushroom samples were smaller than the N content of commercial chitin macromolecules (6.89%). And the mushroom-to-commercial N content ratio varied widely with the type of mushrooms in the range 92.45–42.96%. The reason for low N atom contents in mushroom chitin is that there exists a chitin-glucan complex in mushrooms [9]. Complete removal of glucan does not occur by chemical treatment of the materials that have decreased the N atomic ratio in mushroom chitin. However in the case of crabs and prawns, the matrix substances such as proteins and minerals were completely removed by the chemical treatment with NaOH and HCl solutions (<0.1%) [11]. A similar example of incomplete removal of components is remnants of hemicelluloses in wood [12]. Average fiber widths of chitin nanofibers are listed in Table 1. The width varied depending on the type of mushroom. It is clear from the table that the lower the N content the wider are the nanofibers: 92.45 and 42.96% N containing nanofibers correspond to 20 and 28 nm width, respectively. This indicates that chitin nanofibers with low N content have a certain amount of glucans on the surface of the nanofiber as mentioned above and thus the thickness of the chitin nanofiber is increased.

**Table 1:** Elemental analysis data, crystalline index, fiber width, and yield of chitin nanofibers.

samples	Elemental analysis data			crystalline index	fiber width (nm)	yield (%)
	N (%)	C (%)	N content ratio (%)^a^			
Chitin	6.89	47.29	100	88.5	-	-
Cellulose	0	44.44	0	-	-	-
*Pleurotus eryngii*	6.37	45.43	92.45	80.0	20	3.5
*Agaricus bisporus*	6.19	43.69	89.84	77.1	20	3.2
*Lentinula edodes*	4.89	45.30	70.97	65.0	22	1.3
*Grifola frondosa*	3.67	42.72	53.27	65.0	25	3.2
*Hypsizygus marmoreus*	2.96	43.77	42.96	47.6	28	1.8

^a^ compared with N content of chitin molecule.

**Figure 4:**
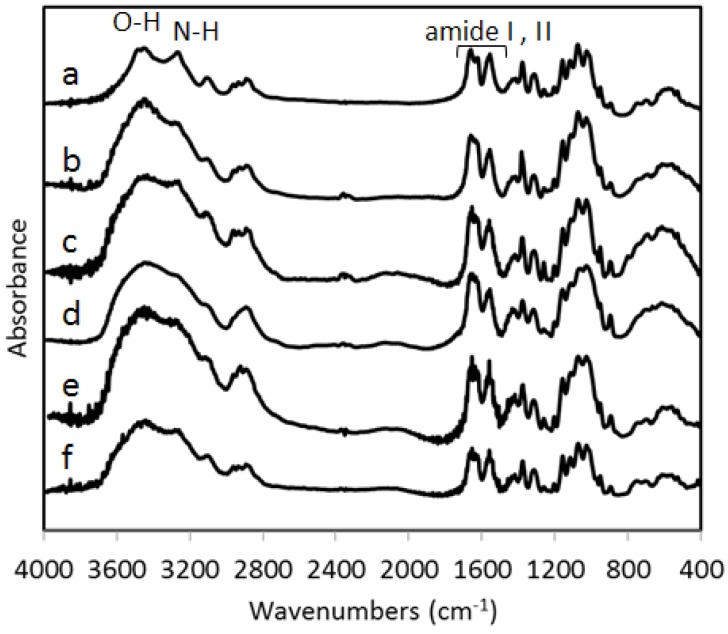
FT-IR spectra of (**a**) commercially available chitin, and chitin nanofibers prepared from (**b**) *Lentinula edodes* (**c**) *Pleurotus eryngii* (**d**) *Hypsizygus marmoreus* (**e**) *Grifola frondosa* and (**f**) *Agaricus bisporus*.

Figure 4 shows the FT-IR spectra (a) of commercial chitin obtained from crab shells and (b–f) are of chitin nanofibers prepared from five species of mushrooms following the method described in this article. The spectral bands (b–f) of chitin nanofibers of mushrooms correspond exactly and overlap with that of commercial pure chitin. The characteristic bands of the chitin molecule: O-H stretching (3450 cm^−1^), N-H stretching (3270 cm^−1^), amide I (1660 and 1620 cm^−1^), and amide II (1560 cm^−1^) are prominent bands observed in the FT-IR spectra of the chitin nanofibers and are similar to the commercial chitin.

**Figure 5:**
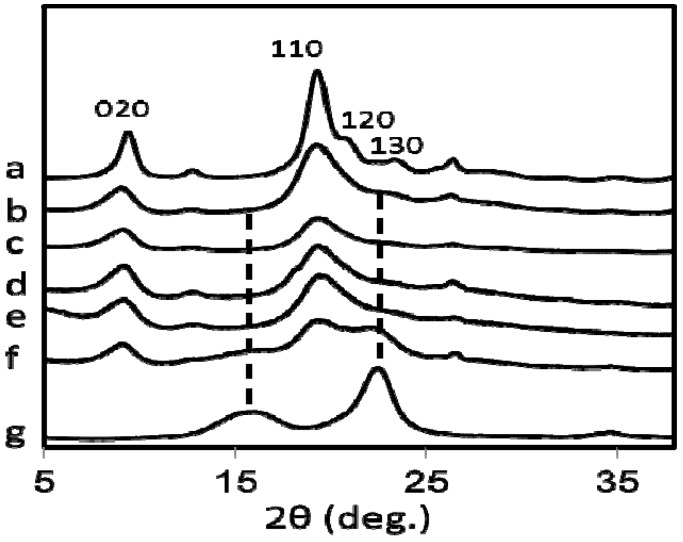
X-ray diffraction profiles of (**a**) commercially available chitin, and chitin nanofibers prepared from (**b**) *Pleurotus eryngii* (**c**) *Agaricus bisporus* (**d**) *Lentinula edodes* (**e**) *Grifola frondosa* (**f**) *Hypsizygus marmoreus* and (**g**) commercially available cellulose.

Figure 5 shows the X-ray diffraction pattern of commercially available chitin (a), (b–f) are the chitin bands of nanofibers prepared from all five species of mushrooms while the pattern (g) is of commercial cellulose. The four diffraction peaks of chitin nanofibers noticed at 9.4°, 19.3°, 20.6°, and 22.5° correspond to the 020, 110, 120, and 130 planes, respectively. They are typical crystal patterns of α-chitin. Thus, chitin nanofibers were extracted from several types of mushroom and the α-chitin crystalline structures were maintained after the removal of matrix substances followed by grinder treatment. However, in the case of *Hypsizygus marmoreus*, which has the lowest N content (Table 1), the X-ray diffractogram (Figure 5f) contains crystal patterns of cellulose (Figure 5g). The diffraction peaks observed from 15° to 17°, and 22.5°, corresponding to the 110, 1–10, and 200 planes, respectively are typical for the cellulose I crystal. Relative crystalline indices (CI) of chitin nanofibers were determined from the X-ray diffraction profiles and listed in Table 1.

Commercial chitin has high crystallinity because the amorphous part is removed by acid hydrolysis in the purification process. Chitin nanofiber from *Pleurotus Eryngii* has the highest CI of 80.0%, which has the highest N content ratio. The indices decreased from 80.0 to 47.6%, with a reasonable correlation to N content ratios from 92.45 to 42.96%, respectively, indicating that the crystallinity also decreased with the increase of the amount of amorphous glucan on the surface of the nanofibers. The CI value would affect the mechanical properties of the chitin nanofiber such as Young’s modulus, fracture strength, and thermal expansion.

## 3. Experimental Section

### 3.1. Materials

*Pleuotus eryngii* (king trumpet mushroom), *Agaricus bisporus* (common mushroom), *Lentinula edodes* (shiitake), *Grifola frondosa* (maitake), and *Hypsizygus marmoreus* (buna-shimeji) were cultivated products of Japan. Chitin powder from crab shells was purchased from Nacalai Tesque. The other chemicals were purchased from Kanto chemical and used as received.

### 3.2. Preparation of Chitin Nanofibers

The five different types of mushroom species were purified to extract the chitin component by a series of chemical treatments based on the method described previously [9]. According to the flowchart shown in Figure 2, first fresh mushrooms (250 g) were coarsely crushed in a domestic blender then were filtered and washed with distilled water to remove the water soluble glucans and minerals. The mashed material was treated with 2% (w/v) NaOH (1 L) for 1day at 100 °C to remove the proteins and alkali soluble glucans. After filtration and washing with distilled water, the residues were treated with 2M HCl (2 L) for 2 days at room temperature to remove residual mineral salts and then washed with distilled water. The residues were mixed with water, 0.5% (w/v) NaClO_2_, and 1% (v/v) AcOH (total 3 L), and the mixtures were stirred at 100 °C for 1 day to bleach the pigment compositions in the sample followed by washing with distilled water. After the extraction with NaClO_2_, the samples were treated again with 2% (w/v) NaOH (1L) at 100 °C for 1 day to remove residual proteins and glucans. After the series of chemical treatments, the samples were filtered and washed with distilled water until the residues were neutralized. The extracted wet chitin was dispersed in water in the concentration of 1 % w/w and acetic acid was added to adjust to pH3. The sample was passed through a grinder (MKCA6-3; Masuko Sangyo Co., Ltd.) at 1500 rpm. Grinder treatment was performed with a clearance gauge of −1.5 (corresponding to a 0.15 mm shift) from the zero position. The position was determined as the point of slight contact between the two grinding stones. The yields of chitin nanofibers thus obtained were calculated based on the dry weight of each species of mushroom chitin nanofibers and the results are listed in Table 1.

### 3.3. Measurements

Infrared spectra of the samples were recorded using potassium bromide pellets with an FT-IR spectrometer (FTIR 8300, Shimadzu). X-ray diffraction profiles of the nanofibers were obtained with Ni-filtered CuKα from an X-ray generator (Ultima IV, Rigaku) operating at 40 kV and 30 mA. The diffraction profile was detected using an X-ray goniometer scanning from 5° to 40°. The crystalline index (CI) was determined by the following equation: CI = (*I*_110_ − *I*_am_) × 100/*I*_110_, where *I*_110_ is the maximum intensity of *I*_110_, and *I*_am_ is the intensity of the amorphous diffraction at 16° [13]. The prepared nanofiber slurry was diluted with EtOH and dried in an oven to prepare a sheet or film of chitin nanofibers. The film was coated with about 2 nm layer of Pt by an ion sputter coater and observed with a field emission scanning electron microscope (JSM-6700F; JEOL, Ltd) operating at 1.5 kV. The average diameter of the isolated nanofibers was estimated counting 30 nanofibers manually.

## 4. Conclusions 

Chitin nanofibers were prepared from the cell wall of a number of edible mushrooms by a series of chemical and grinding treatments. The chitin nanofibers thus obtained have uniform structures. Depending on the species of mushroom, the width of chitin nanofibers varied in the range 20–28 nm. The simple method developed enabled the manufacture of homogeneous chitin nanofibers which are important dietary fibers. The dietary nanofibers thus obtained had very long fiber length, high dispersibility in water, and high viscosity. It was noticed that the chitin nanofibers formed a complex with several glucans on the surface of the fibers. As polysaccharides, the chitin nanofibers with special physical properties can have antitumor application and immuno-modulating activity. With the development of our mushroom nanofiber research, in addition to crab and prawn shell nanofibers, the mushroom nanofibers have now been added to the list as functional food ingredients as well as useful for medical applications.
